# Impact of *PSCA* Polymorphisms on the Risk of Duodenal Ulcer

**DOI:** 10.2188/jea.JE20190184

**Published:** 2021-01-05

**Authors:** Yoshiaki Usui, Keitaro Matsuo, Isao Oze, Tomotaka Ugai, Yuriko Koyanagi, Yoshinobu Maeda, Hidemi Ito, Asahi Hishida, Kenji Takeuchi, Takashi Tamura, Mineko Tsukamoto, Yuka Kadomatsu, Megumi Hara, Yuichiro Nishida, Ippei Shimoshikiryo, Toshiro Takezaki, Etsuko Ozaki, Daisuke Matsui, Isao Watanabe, Sadao Suzuki, Miki Watanabe, Hiroko Nakagawa-Senda, Haruo Mikami, Yohko Nakamura, Kokichi Arisawa, Hirokazu Uemura, Kiyonori Kuriki, Naoyuki Takashima, Aya Kadota, Hiroaki Ikezaki, Masayuki Murata, Masahiro Nakatochi, Yukihide Momozawa, Michiaki Kubo, Kenji Wakai

**Affiliations:** 1 Division of Cancer Information and Control, Department of Preventive Medicine, Aichi Cancer Center, Nagoya, Japan; 2 Department of Hematology and Oncology, Okayama University Graduate School of Medicine, Dentistry and Pharmaceuticals Sciences, Okayama, Japan; 3 Division of Cancer Epidemiology and Prevention, Department of Preventive Medicine, Aichi Cancer Center, Nagoya, Japan; 4 Department of Epidemiology, Nagoya University Graduate School of Medicine, Nagoya, Japan; 5 Division of Descriptive Cancer Epidemiology, Nagoya University Graduate School of Medicine, Nagoya, Japan; 6 Department of Preventive Medicine, Nagoya University Graduate School of Medicine, Nagoya, Japan; 7 Department of Preventive Medicine, Faculty of Medicine, Saga University, Saga, Japan; 8 Department of International Island and Community Medicine, Kagoshima University Graduate School of Medical and Dental Sciences, Kagoshima, Japan; 9 Department of Epidemiology for Community Health and Medicine Kyoto Prefectural University of Medicine, Kyoto, Japan; 10 Department of Public Health, Nagoya City University Graduate School of Medical Sciences, Nagoya, Japan; 11 Cancer Prevention Center, Chiba Cancer Center Research Institute, Chiba, Japan; 12 Department of Preventive Medicine, Institute of Biomedical Sciences, Tokushima University Graduate School, Tokushima, Japan; 13 Laboratory of Public Health, University of Shizuoka, Shizuoka, Japan; 14 Department of Public Health, Faculty of Medicine, Kindai University, Osaka, Japan; 15 Department of Public Health, Shiga University of Medical Science, Otsu, Japan; 16 Department of General Internal Medicine, Kyushu University Hospital, Fukuoka, Japan; 17 Department of Nursing, Nagoya University Graduate School of Medicine, Nagoya, Japan; 18 Laboratory for Genotyping Development, RIKEN Center for Integrative Medical Sciences, Yokohama, Japan

**Keywords:** *PSCA*, duodenal ulcer, cross-sectional study, Japan

## Abstract

**Background:**

While duodenal ulcer (DU) and gastric cancer (GC) are both *H. pylori* infection-related diseases, individuals with DU are known to have lower risk for GC. Many epidemiological studies have identified the *PSCA* rs2294008 T-allele as a risk factor of GC, while others have found an association between the rs2294008 C-allele and risk of DU and gastric ulcer (GU). Following these initial reports, however, few studies have since validated these associations. Here, we aimed to validate the association between variations in *PSCA* and the risk of DU/GU and evaluate its interaction with environmental factors in a Japanese population.

**Methods:**

Six *PSCA* SNPs were genotyped in 584 DU cases, 925 GU cases, and 8,105 controls from the Japan Multi-Institutional Collaborative Cohort (J-MICC). Unconditional logistic regression models were applied to estimate odds ratios (ORs) and 95% confidence intervals (CIs) for the association between the SNPs and risk of DU/GU.

**Results:**

*PSCA* rs2294008 C-allele was associated with per allele OR of 1.34 (95% CI, 1.18–1.51; *P* = 2.28 × 10^−6^) for the risk of DU. This association was independent of age, sex, study site, smoking habit, drinking habit, and *H. pylori* status. On the other hand, we did not observe an association between the risk of GU and *PSCA* SNPs.

**Conclusions:**

Our study confirms an association between the *PSCA* rs2294008 C-allele and the risk of DU in a Japanese population.

## INTRODUCTION

Peptic ulcer—duodenal ulcer (DU) and gastric ulcer (GU)—is one of the most common gastrointestinal diseases, with an estimated lifetime prevalence of 5–10% in the general population.^1^ Peptic ulcer is defined as a mucosal defect which penetrates through the muscularis mucosa with a diameter of at least 0.5 cm.^2^ Among the various risk factors of peptic ulcer reported to date, including smoking and drinking, one of the main factors is infection with *Helicobacter pylori* (*H. pylori*),^3^^,^^4^ which is an established risk factor of gastric cancer.^5^

**Figure 1.**
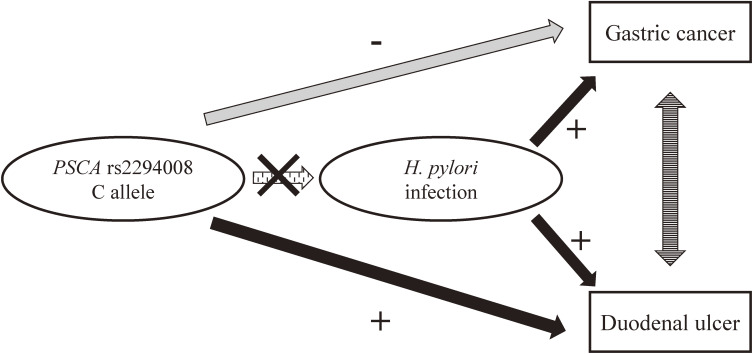
Association between duodenal ulcer/gastric cancer and host factors (*PSCA* rs2294008). *H. pylori* infection increased the risk of both gastric cancer and duodenal ulcer. On the other hand, *PSCA* rs2294008 C-allele decreased the risk of gastric cancer and increased that of duodenal ulcer.

While both DU and gastric cancer (GC) are *H. pylori* infection-related diseases, individuals with DU are well known to have a lower risk for GC.^6^^,^^7^ It is increasingly clear that these heterogeneities are influenced by not only bacterial but also host factors. A genome-wide association study (GWAS) found an association between *PSCA* rs2294008 T-allele and the risk of GC (per allele OR 1.67, *P* value 2.2 × 10^−15^) in a Japanese population,^8^ while a second GWAS found associations between variations in *PSCA* gene and *ABO* gene and the risk of DU in an Japanese population.^9^ These latter authors reported that the *PSCA* rs2294008 C-allele increased the risk of DU (per allele OR 1.84, *P* value 3.92 × 10^−33^) but decreased that of GC (per allele OR 0.79, *P* value 6.79 × 10^−12^).^9^ In addition, they reported that the *PSCA* rs2294008 C-allele increased the risk of GU (per allele OR 1.13, *P* value 5.85 × 10^−7^); however, *ABO* polymorphisms are not significantly associated with the risk of GU.^10^ They also reported the function relevance of *PSCA* for peptic ulcer and gastric cancer^8^^,^^9^; on the other hand, the function relevance of *ABO* for peptic ulcer has not been clarified yet. The association between DU/GC and these risk factors is summarized in Figure 1. Following these initial reports, however, few studies have since validated this association.

Here, we conducted a cross-sectional study to replicate the association between the variations in the previously reported *PSCA* loci^9^^,^^10^ and risk of DU/GU, and evaluate the interaction between these variations and smoking/drinking status and *H. pylori* status on the risk of DU/GU in a Japanese population. We also evaluated the *ABO* loci, as reported in two previous studies.^9^^,^^10^

## MATERIAL AND METHODS

### Study subjects

**Figure 2.**
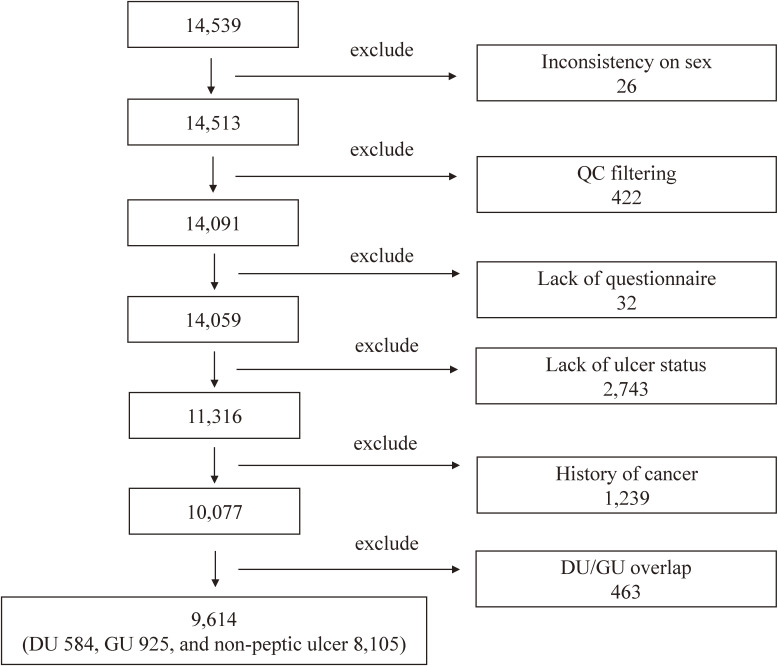
Study subject selection. A total of 14,539 participants were randomly selected for genotyping from 47,163 participants. We excluded 26 subjects because of inconsistent baseline information between the questionnaire and genotyping on sex; 422 whose genotype data did not meet quality control (QC) filters; 32 because of a lack of questionnaire data; 2,743 with a history of cancer; 1,239 with a lack of data on ulcer status; and 463 with DU/GU overlap cases. Finally, we selected 9,614 subjects for participation in this study. QC, quality control; DU, duodenal ulcer; GU, gastric ulcer

The Japan Multi-Institutional Collaborative Cohort (J-MICC) study is a large cohort study launched in 2005 to confirm and detect gene-environment interactions in lifestyle-related disease. Details of the J-MICC study have been reported elsewhere.^11^ Briefly, the study includes 92,647 participants aged 35–69 years from 13 areas throughout Japan (Aichi, Chiba, Fukuoka, Iga, Kagoshima, Kyushu-KOPS, Kyoto, Okazaki, Sakuragaoka, Saga, Shizuoka-Daiko, Takashima and Tokushima sub-cohorts) as at end of March 2014. All participants gave written informed consent to participate; answered a questionnaire that inquired about lifestyle-related factors, past medical history, medication status and anthropometric characteristics; and provided a blood sample. The study protocol was approved by the Ethics Committees of Nagoya University Graduate School of Medicine and the other institutions participating in the J-MICC study. The present study was conducted in accordance with the principles expressed in the World Medical Association Declaration of Helsinki. A total of 14,539 participants were randomly selected to be genotyped from 47,163 participants in 12 areas (except for the Iga sub-cohort, where the survey was conducted from 2013 to 2014) recruited between 2004 and 2013. Subjects were selected as shown in Figure 2. We excluded 26 subjects because of inconsistent baseline information between the questionnaire and genotyping on sex; 422 whose genotype data did not meet quality control (QC) filters; 32 because of a lack of questionnaire data; 2,743 with a history of cancer; and 1,239 with a lack of data on ulcer status. In addition, to clarify the substantial impact on the risk of DU or the risk of GU, we excluded 463 with DU/GU overlap cases. Finally, we selected 9,614 subjects for participation in this study.

### Past medical history and lifestyle-related factors

The questionnaire for the J-MICC study included questions on past medical history, and cigarette smoking, alcohol drinking, and coffee drinking habits. Medical histories for DU and GU were enquired about in the three categories of never, past, and current. The combination of a past and current medical history of DU/GU was considered positive, and otherwise as negative. Smoking/drinking habits were enquired about in the three categories of never, former, and current. Former smokers/drinkers were defined as those who had quit smoking/drinking for more than 1 year. Never smokers were defined as those who smoked less than 100 cigarettes in their lifetime. We defined the combination of former and current smokers/drinkers as ever smokers/drinkers. Smoking habit was evaluated in pack-years, calculated by multiplying the number of packs consumed per day by the number of years of smoking. Alcohol consumption of each beverage type (Japanese sake, beer, shochu, whiskey, and wine) was estimated as the average number of drinks per day, which was converted into a Japanese sake equivalent. One “go” of Japanese sake contains 23 g of ethanol, which is equal to one large bottle (633 mL) of beer, 108 mL of shochu (distilled spirit), two shots (57 mL) of whiskey, or two and a half glasses of wine (200 mL). Total alcohol consumption was determined as the total sum of pure ethanol consumption (g/day) of each alcohol beverage. Coffee consumption was obtained in terms of the frequency and amount of cups according to the following categories: almost none, 1–2 cups/week, 3–4 cups/week, 5–6 cups/week, 1–2 cups/day, 3–4 cups/day, and ≥5 cups/day. We classified coffee consumption based on its distribution among the subjects as almost none, <1 cup of day, and ≥1 cup of day. This study is based on the data version J-MICC_CS_20180111.

### Genotyping and quality control filtering

DNA was prepared from buffy coat fractions using a BioRobot M48 Workstation (Qiagen Group, Tokyo, Japan) at the central study office. For the samples from two areas (Fukuoka and Kyushu-KOPS), DNA was extracted from samples of whole blood using an automatic nucleic acid isolation system (NA-3000; Kurabo, Osaka, Japan). Genotyping for all 14,539 study participants from the 12 areas of the J-MICC Study was done at the RIKEN Center for Integrative Sciences using a HumanOmniExpressExome-8 v1.2 Bead Chip array (Illumina Inc., San Diego, CA, USA). The 26 samples with inconsistent sex information between the questionnaire and genotyping results were excluded. The identity-by-descent method implemented in the PLINK 1.9 software^12^ found 388 close relationship pairs (pi-hat > 0.1875) and one sample of each pair were excluded. Principal component analysis^13^^,^^14^ with the 1,000 Genomes reference panel (phase 3)^15^ detected 34 subjects whose estimated ancestries were non-Japanese,^16^ and these were also excluded. The remaining 14,091 samples all met the sample-wise genotype call rate criterion (≥0.99). Single nucleotide polymorphisms (SNPs) with a genotype call rate <0.98, a Hardy-Weinberg equilibrium exact test *P* value <1 × 10^−6^, a minor allele frequency of <0.01, or a departure from the allele frequency computed from the 1,000 Genomes Project phase 3 EAS samples were removed. Non-autosomal SNPs were also removed. This QC filtering resulted in 14,091 samples and 570,162 autosomal variants.

### Genotype imputation

Genotype imputation was performed using SHAPEIT^17^ and Minimac3^18^ software base on the 1,000 Genomes reference panel (phase 3). After genotype imputation, variants with an imputation quality r^2^ < 0.3 were excluded, resulting in 12,617,547 variants. In our primary analysis, we used imputed genotype data, namely GT format output by Minimac3, estimation of most likely genotype. We also evaluated allele dosage data by imputation as well to evaluate consistency with genotype data analysis.

### Candidate SNP selection

To reduce the number of SNPs tested in this analysis, we prespecified tagSNPs based on HapMap-JPT data using National Institute of Health (NIH) LD TAG SNP Selection.^19^ We selected *PSCA* six SNPs and *ABO* 18 SNPs from eFigure 1 and eFigure 2. We applied an R^2^ threshold of 0.8 for SNPs with a MAF of more than 0.05. We forced the inclusion of previously reported SNPs (*PSCA* rs2294008 and *ABO* rs505922) due their association with the risk of DU in a previous study.^9^ In addition, we assessed accordance with Hardy-Weinberg equilibrium using the chi-squared test.

### Serum sample measurement

We also evaluated *H. pylori* status by measuring anti-*H. pylori* IgG serum antibody in 2,760 samples from four among twelve study sites of the J-MICC Study (Daiko, Kyoto, Aichi Cancer Center, and Okazaki). Serum samples were immediately stored at −80°C until measurement. Anti-*H. pylori* IgG serum antibody was measured using a direct ELISA kit, “E plate ‘Eiken’ *H. pylori* Antibody” (Eiken Kagaku, Tokyo, Japan), with values of 10.0 units/mL or higher regarded as seropositive according to the manufacturer’s instructions.

### Statistical analysis

First, to narrow down the number of SNPs for interaction analysis, we analyzed the association of the SNPs with the risk of DU/GU by unconditional logistic regression analysis adjusted for age (continuous), sex, and study site. We also analyzed the association of the SNPs with the risk of *H. pylori* infection by the same model. We applied Bonferroni corrected *P* values of 0.05/24 to avoid false positive associations. Second, we examined for interaction between selected SNP and smoking/drinking status for the risk of DU/GU. We included interaction term between minor allele numbers (0, 1, and 2) of corresponding SNP and status (ever vs never) in the models. We added pack-years for ever smokers and total sum of pure alcohol consumption (g/day) for ever drinkers as covariates in the models. Finally, we examined interaction between selected SNP and *H. pylori* status for the risk of DU/GU among available data. We included interaction term between the minor allele numbers (0, 1, and 2) of corresponding SNP and *H. pylori* status. Throughout the analysis, we estimated per allele odds ratios (ORs) and their 95% confidence intervals (CIs) using the major allele homozygote as reference. All analyses were performed using STATA version 15.1 software (Stata Corp., College Station, TX, USA).

## RESULTS

Our analysis included 584 DU cases, 925 GU cases, and 8,105 controls. Table 1 summarizes the demographic, lifestyle, and medical characteristics of the study subjects by DU/GU status. Mean age was 56.2 years in DU, 56.6 years in GU, and 53.1 years in the controls, respectively. The proportion of males was higher in the case groups than in the control group (63.4% in DU, 57.6% in GU, and 41.5% in the controls). The proportion of never drinkers was lower in the case groups than in the control group (30.3% in DU, 36.5% in GU, and 42.1% in the controls). Similarly, the proportion of never smokers was lower in the case groups than in the control group (40.4% in DU, 44.5% in GU, and 62.8% in the controls). Although *H. pylori* status data was only available for some participants, the proportion who were *H. pylori* status-positive was higher in the case groups than in the control group (12.0% in DU, 13.3% in GU, and 9.6% in the controls).

**Table 1.** Characteristics of the study subjects

	Duodenal ulcer (*n* = 584)	Gastric ulcer (*n* = 925)	Non-peptic ulcer (*n* = 8,105)
Age, years (%)			
<40	19 (3.25)	26 (2.81)	847 (10.45)
40–49	130 (22.26)	175 (18.92)	2,174 (26.82)
50–59	198 (33.90)	321 (34.70)	2,619 (32.31)
60–69	237 (40.58)	403 (43.57)	2,465 (30.41)
Mean (SD)	56.16 (8.66)	56.60 (8.31)	53.08 (9.62)

BMI, kg/m^2^ (%)			
<21	145 (24.83)	255 (27.57)	2,191 (27.03)
≥21, <23	141 (24.14)	284 (30.70)	2,129 (26.27)
≥23, <25	144 (24.66)	185 (20.00)	1,823 (22.49)
≥25	152 (26.03)	192 (20.76)	1,885 (23.26)
Unknown	2 (0.34)	9 (0.97)	77 (0.95)
Mean (SD)	23.30 (3.19)	22.81 (3.10)	23.03 (3.28)

Sex (%)			
Male	370 (63.36)	533 (57.62)	3,365 (41.52)
Female	214 (36.64)	392 (42.38)	4,740 (58.48)

Drinking status (%)			
Never	177 (30.31)	338 (36.54)	3,415 (42.13)
Former drinker	16 (2.74)	20 (2.16)	144 (1.41)
Current drinker	390 (66.78)	567 (61.30)	4,574 (56.43)
Unknown	1 (0.17)	0 (0.00)	2 (0.02)

Amount of drinking (%)			
0	202 (34.59)	371 (40.11)	3,634 (44.84)
<23 g/day	189 (32.36)	272 (29.41)	2,686 (33.14)
≥23, <46 g/day	87 (14.90)	119 (12.86)	761 (9.39)
≥46 g/day	77 (13.18)	119 (12.86)	705 (8.70)
Unknown	29 (4.97)	44 (4.76)	319 (3.94)
Mean (SD)	17.96 (25.79)	17.20 (27.21)	12.89 (23.83)

Smoking status (%)			
Never	236 (40.41)	412 (44.54)	5,089 (62.79)
Former smoker	168 (28.77)	237 (25.62)	1,455 (17.95)
Current smoker	180 (30.82)	276 (29.84)	1,557 (19.21)
Unknown	0 (0.00)	0 (0.00)	4 (0.05)

Pack-years (%)			
0	237 (40.58)	412 (44.54)	5,105 (62.99)
>0, <20	110 (18.84)	149 (16.11)	1,271 (15.68)
≥20	233 (39.90)	358 (38.70)	1,676 (20.68)
Unknown	4 (0.68)	6 (0.65)	53 (0.65)
Mean (SD)	18.47 (23.22)	18.90 (24.92)	9.95 (18.47)

Coffee consumption (%)			
almost none	114 (19.52)	165 (17.84)	1,310 (16.16)
>0, 1> cup of day	158 (27.05)	269 (29.08)	2,224 (27.44)
≥1 cup of day	309 (52.91)	491 (53.08)	4,550 (56.14)
Unknown	0 (0.00)	0 (0.00)	21 (0.26)

*H. pylori* status^a^ (%)			
Negative	80 (13.70)	174 (18.81)	1,533 (18.91)
Positive	70 (11.99)	123 (13.30)	780 (9.62)
Data unavailable	434 (74.32)	628 (67.89)	5,792 (71.46)

^a^*H. pylori* status was evaluated by measuring anti-*H. pylori* IgG antibody for 2,760 available samples.

Negative: IgG <10.0 unit/mL, Positive: IgG ≥10.0 unit/mL.

eTable 1 shows allele frequencies of *PSCA* and *ABO* SNPs at the survey; the r^2^ at the imputation; and MAF in the HapMap-JPT data set, Human Genetic Variation Database (HGVD)^20^ and Integrative Japanese Genome Variation Database (IJGVD).^21^ All SNPs were in accordance with the Hardy-Weinberg equilibrium. Imputation quality for all SNPs showed high accuracy (r^2^ > 0.8). All SNPs showed a difference in MAF of less than 0.1 between this survey and the HapMap JPT dataset, HGVD or IJGVD.^21^

The association of *PSCA* and *ABO* SNPs with the risk of DU and GU is shown in Table 2. The *PSCA* polymorphisms were significantly associated with the risk of DU (rs2294008, rs2920296, and rs2976397), while *PSCA* polymorphisms were not associated with the risk of GU. The *ABO* polymorphisms were not associated with the risk of DU or GU. We also analyzed data of allele dosage imputed by Minimac3,^18^ and consistent results were observed (as shown in eTable 2). For *PSCA* rs2294008, the *P* value was 2.28 × 10^−6^ for DU (per allele OR 1.34; 95% CI, 1.18–1.51). Rs2920296 and rs2976397 had also significant *P* values, and rs2920296 had the lowest *P* value, but we did not choose rs2920296 and rs2976397 for further detailed analysis based on the fact rs2294008 is the truly functional locus.^9^

**Table 2.** Association of *PSCA* and *ABO* SNPs with risk of duodenal ulcer and gastric ulcer

Gene	rs number	Chr	Position	Allele A/a^a^	Control			Dudenal ulcer						Gastric ulcer					
		
					Genotype prevalence cases (*n* = 1,047)			Genotype prevalence controls (*n* = 9,030)			Per allele OR^b^	95% CI^b^	*P* value^b^	Genotype prevalence controls (*n* = 8,689)			Per allele OR^b^	95% CI^b^	*P* value^b^
		
					AA	Aa	aa	AA	Aa	aa				AA	Aa	aa			
*PSCA*	rs6471587	8q24	143761103	C/G	0.741	0.242	0.017	0.755	0.224	0.021	0.95	0.80–1.14	6.07E-01	0.734	0.250	0.016	1.02	0.89–1.18	7.58E-01
*PSCA*	rs2294008	8q24	143761931	T/C	0.370	0.474	0.156	0.320	0.428	0.252	1.34	1.18–1.51	**2.28E-06**	0.336	0.502	0.162	1.08	0.98–1.20	1.11E-01
*PSCA*	rs2976391	8q24	143762724	C/A	0.654	0.309	0.037	0.610	0.324	0.067	1.23	1.07–1.43	4.01E-03	0.655	0.311	0.034	0.98	0.86–1.11	7.37E-01
*PSCA*	rs3736001	8q24	143762807	G/A	0.802	0.187	0.011	0.801	0.187	0.012	1.01	0.83–1.23	9.01E-01	0.803	0.186	0.011	0.99	0.84–1.16	8.68E-01
*PSCA*	rs2920296	8q24	143763109	G/A	0.369	0.475	0.156	0.319	0.430	0.252	1.34	1.19–1.51	**1.83E-06**	0.336	0.502	0.162	1.08	0.98–1.20	1.14E-01
*PSCA*	rs2976397	8q24	143764613	G/T	0.286	0.495	0.219	0.385	0.437	0.178	0.76	0.67–0.85	**7.77E-06**	0.304	0.508	0.188	0.91	0.83–1.01	6.44E-02

*ABO*	rs8176749	9q34	136131188	C/T	0.691	0.281	0.028	0.687	0.281	0.033	1.03	0.88–1.21	6.78E-01	0.714	0.265	0.022	0.89	0.78–1.02	1.07E-01
*ABO*	rs8176747	9q34	136131315	C/G	0.680	0.290	0.030	0.683	0.274	0.043	1.03	0.88–1.21	6.82E-01	0.706	0.271	0.023	0.88	0.77–1.01	6.47E-02
*ABO*	rs8176740	9q34	136131472	A/T	0.538	0.390	0.071	0.555	0.365	0.081	0.99	0.86–1.13	8.89E-01	0.552	0.386	0.062	0.95	0.85–1.07	4.19E-01
*ABO*	rs7853989	9q34	136131592	G/C	0.674	0.294	0.032	0.676	0.281	0.043	1.03	0.88–1.20	7.30E-01	0.700	0.276	0.025	0.89	0.78–1.01	7.44E-02
*ABO*	rs8176731	9q34	136132350	T/C	0.305	0.492	0.203	0.303	0.491	0.206	1.01	0.90–1.14	8.17E-01	0.337	0.482	0.181	0.90	0.82–1.00	4.07E-02
*ABO*	rs8176725	9q34	136132617	G/A	0.533	0.394	0.073	0.531	0.389	0.081	1.00	0.88–1.15	9.69E-01	0.551	0.380	0.069	0.93	0.83–1.04	1.79E-01
*ABO*	rs8176722	9q34	136132754	C/A	0.668	0.299	0.033	0.670	0.284	0.046	1.04	0.89–1.21	6.24E-01	0.693	0.282	0.025	0.89	0.78–1.01	7.25E-02
*ABO*	rs8176720	9q34	136132873	T/C	0.308	0.491	0.201	0.307	0.491	0.202	1.01	0.90–1.14	8.60E-01	0.345	0.479	0.176	0.89	0.81–0.98	2.05E-02
*ABO*	rs512770	9q34	136133506	G/A	0.542	0.387	0.071	0.560	0.361	0.079	0.98	0.86–1.13	8.25E-01	0.562	0.376	0.062	0.94	0.84–1.05	2.91E-01
*ABO*	rs549446	9q34	136135238	C/T	0.538	0.389	0.073	0.557	0.363	0.081	0.99	0.86–1.13	8.28E-01	0.560	0.378	0.062	0.94	0.84–1.05	2.41E-01
*ABO*	rs493211	9q34	136136516	G/A	0.538	0.389	0.072	0.557	0.363	0.081	0.99	0.86–1.13	8.31E-01	0.560	0.378	0.062	0.94	0.84–1.05	2.42E-01
*ABO*	rs688976	9q34	136136770	C/A	0.538	0.389	0.073	0.557	0.363	0.081	0.99	0.86–1.13	8.28E-01	0.560	0.378	0.062	0.94	0.84–1.05	2.41E-01
*ABO*	rs2073828	9q34	136137140	G/A	0.525	0.394	0.081	0.533	0.377	0.091	1.01	0.89–1.16	8.28E-01	0.506	0.409	0.085	1.07	0.96–1.19	2.39E-01
*ABO*	rs8176694	9q34	136137646	T/C	0.775	0.210	0.015	0.781	0.200	0.019	0.98	0.82–1.18	8.65E-01	0.797	0.192	0.011	0.88	0.75–1.03	1.20E-01
*ABO*	rs514659	9q34	136142203	A/C	0.306	0.489	0.205	0.322	0.444	0.235	1.01	0.89–1.14	8.96E-01	0.309	0.475	0.216	0.99	0.90–1.10	8.97E-01
*ABO*	rs500498	9q34	136148647	C/T	0.304	0.488	0.208	0.344	0.444	0.212	0.94	0.84–1.06	3.43E-01	0.314	0.494	0.192	0.97	0.88–1.07	4.85E-01
*ABO*	rs505922	9q34	136149229	T/C	0.298	0.493	0.209	0.315	0.444	0.241	1.02	0.90–1.15	7.94E-01	0.306	0.475	0.220	0.99	0.89–1.09	7.93E-01
*ABO*	rs630014	9q34	136149722	G/A	0.393	0.471	0.137	0.408	0.449	0.144	1.00	0.88–1.13	9.90E-01	0.405	0.451	0.144	1.01	0.91–1.12	8.32E-01

^a^Allele A, major allele; allele a, minor allele.

^b^For additive models, gender-, age- and site-adjusted per allele OR; 95% CI and *P* values calculated by logistic regression are shown. Major alleles were considered as references. *P* values <2.08E-03 (0.05/24) are highlighted in boldface.

Threshold was Bonferroni significance.

The association of *PSCA* rs2294008 with the risk of DU/GU stratified by smoking/drinking status is shown in Table 3. *PSCA* rs2294008 was significantly associated with the risk of DU regardless of smoking status. Similarly, after stratification by drinking status, *PSCA* rs2294008 was also significantly associated with the risk of DU. Regarding GU, we did not observe any association with *PSCA* rs2294008 after stratification by smoking/drinking status. We did not observe obvious multiplicative interaction between *PSCA* rs2294008 and smoking/drinking status for the risk of DU/GU. We also examined interaction between the selected *PSCA* rs2294008 and coffee consumption for the risk of DU/GU, but again saw no obvious multiplicative interaction (data not shown). Addition of pack-years for ever smokers and g/day for ever drinkers as covariates resulted in no significant change in point estimates (per allele OR 1.29; 95% CI, 1.10–1.51 in ever smokers; per allele OR 1.33; 95% CI, 1.15–1.55 in ever drinkers).

**Table 3.** Association of *PSCA* rs2294008 with the risk of duodenal ulcer and gastric ulcer stratified by smoking and drinking status

Disease			Ever^c^						Never						*P* value for interaction^e^
	
			TT	CT	CC	Per allele OR^d^	95% CI^d^	*P* value^d^	TT	CT	CC	Per allele OR^d^	95% CI^d^	*P* value^d^	
Duodenal ulcer^a^															
	Smoking	Cases	121	135	92	1.28	1.10–1.50	**1.78E-03**	66	115	55	1.41	1.17–1.70	**2.98E-04**	0.509
		Controls	1,108	1,427	477				1,885	2,417	787				
															
	Drinking	Cases	130	170	106	1.33	1.15–1.54	**1.08E-04**	57	79	41	1.32	1.06–1.64	**1.34E-02**	0.975
		Controls	1,725	2,207	756				1,269	1,636	510				

Gastric ulcer^b^															
	Smoking	Cases	178	246	89	1.09	0.95–1.24	2.37E-01	133	218	61	1.08	0.93–1.25	3.26E-01	0.953
		Controls	1,108	1,427	477				1,885	2,417	787				
															
	Drinking	Cases	193	298	96	1.08	0.96–1.23	2.06E-01	118	166	54	1.08	0.91–1.27	3.83E-01	0.934
		Controls	1,725	2,207	756				1,269	1,636	510				

^a^We analyzed 584 duodenal ulcer cases and 8,105 controls.

^b^We analyzed 925 gastric ulcer cases and 8,105 controls.

^c^Ever smoker: former smoker and current smoker. Ever drinker: former drinker and current drinker.

^d^Gender-, age- and site-adjusted per allele OR; 95% CI and *P* values calculated by logistic regression are shown. Major alleles were considered as references. *P* values <0.05 are highlighted in boldface.

^e^Interaction between *PSCA* rs2294008 and smoking/drinking was evaluated by logistic regression model including an interaction term between smoking/drinking and *PSCA* rs2294008.

We did not observe an association of *PSCA* and *ABO* SNPs and the risk of *H. pylori* infection (as shown in eTable 3). The association of *PSCA* rs2294008 with the risk of DU/GU stratified by *H. pylori* status among available data is shown in Table 4. This analysis included 150 DU cases, 297 GU cases, and 2,313 controls. *PSCA* rs2294008 was significantly associated with the risk of DU regardless of *H. pylori* status. Regarding GU, we did not observe significant association with *PSCA* rs2294008 after stratification by *H. pylori* status. We did not observe obvious multiplicative interaction between *PSCA* rs2294008 and *H. pylori* status for the risk of DU/GU.

**Table 4.** Association of *PSCA* rs2294008 with risk of duodenal ulcer and gastric ulcer stratified by *H. pylori* status

Disease		*H. pylori* status^c^												*P* value for interaction^e^

		Negative						Positive						
	
		TT	CT	CC	Per allele OR^d^	95% CI^d^	*P* value^d^	TT	CT	CC	Per allele OR^d^	95% CI^d^	*P* value^d^	
Duodenal ulcer^a^	Cases	23	34	23	1.47	1.08–2.02	**1.50E-02**	20	27	23	1.57	1.12–2.21	**9.00E-03**	0.802
	Controls	572	701	260				281	362	137				

Gastric ulcer^b^	Cases	53	99	22	1.05	0.84–1.31	6.75E-01	45	55	23	1.03	0.79–1.36	8.06E-01	0.858
	Controls	572	701	260				281	362	137				

^a^We analyzed 150 duodenal ulcer cases and 2,313 controls.

^b^We analyzed 297 gastric ulcer cases and 2,313 controls.

^c^*H. pylori* status was defined by anti-*H. pylori* serum IgG antibody. Negative: IgG <10.0 unit/mL, Positive: IgG ≥10.0 unit/mL.

^d^Gender-, age- and site-adjusted per allele OR; 95% CI and *P* values calculated by logistic regression are shown. Major alleles were considered as references. *P* values <0.05 are highlighted in boldface.

^e^Interaction between *PSCA* rs2294008 and *H. pylori* status was evaluated by logistic regression model including an interaction term between *H. pylori* status and *PSCA* rs2294008.

## DISCUSSION

We found a significant association between variations in *PSCA* and risk of DU. This association was consistent regardless of age, sex, and study site. However, we did not find an association between variations in *PSCA* and the risk of GU and an association between variations in *ABO* and risk of DU/GU. After stratification of the environmental factors smoking/drinking and *H. pylori* status, *PSCA* rs2294008 was also significantly associated with the risk of DU. No obvious multiplicative interaction between *PSCA* rs2294008 and smoking/drinking and *H. pylori* status was observed.

Our study suggests that *PSCA* rs2294008 C-allele was associated with an increased risk of DU in the Japanese population. This is consistent with previous studies.^9^^,^^22^ The *PSCA* gene is located on chromosome 8q24.2 and encodes a cell membrane glycoprotein which belongs to the Thy-1/Ly-6 family. Several reports suggest that this glycoprotein is involved in cell renewal and proliferation.^23^^–^^25^ While overexpressed in some types of cancers, including prostate, bladder, and pancreatic cancer,^23^^,^^26^^,^^27^ it is downregulated in esophageal and gastric cancer.^28^ Functional analysis in a previous GWAS revealed a considerable function for *PSCA* rs2294008. Tanikawa et al reported that PSCA protein encoded by the rs2294008 T-allele with an additional fragment of nine amino acids at the N-terminus and its localization changes from the cytoplasm to the cell surface, whereas short PSCA protein encoded by the rs2294008 C-allele is localized to the cytoplasm. They also suggested that the shorter PSCA protein encoded by the rs2294008 C-allele might result in insufficient epithelial proliferation to counteract the damage due to a lack of functional cell surface PSCA, resulting in slow recovery from duodenal tissue damage.^9^ Those reports may support a significant association between the *PSCA* rs2294008 C-allele and risk of DU.

In contrast with DU, we did not observe the association between *PSCA* polymorphisms and the risk of GU. Although it is difficult to completely rule out the possibility of lack of statistical power, but our result indicates that *PSCA* polymorphisms have higher impact on the risk of DU than on the risk of GU. This difference in the magnitude of association by site is consistent with the previous reports.^9^^,^^10^ In general, DU and GU have a different etiological spectrum, and differ with regard to the severity and distribution of background gastritis.^29^ DU is usually diagnosed in patients with high antral inflammatory scores and high acid secretion, whereas GU is diagnosed in patients with corporal gastritis or pan-gastritis and with normal or decreased acid secretion. These differences might be related to the different impact of these *PSCA* polymorphisms on the risk of DU and GU. Further clarification of the biological mechanism is required.

In addition, in contrast with previous reports,^9^ we did not observe the association between *ABO* polymorphisms and the risk of DU. One of the reason for this discrepancy might be difference of study population between previous study and this study. Previous study employed subjects from individuals with 47 diseases at the hospital.^9^ On the other hand, this study was population-based cohort study mostly from the general population.^11^ Further evaluation in different population is needed.

This study had several strengths. First, to our knowledge, it is the largest replication studies of this association in an Asian population following the initial report. Second, it is the first study to evaluate the interaction between *PSCA* rs2294008 and smoking/drinking and *H. pylori* status on the risk of DU/GU. Finally, although it is said that candidate gene approach tends to have greater statistical power than GWAS,^30^ we did not observe previously reported associations.

Several limitations of this study should also be mentioned. First, it was based on a cross-sectional study in which exposure and outcome measurements were performed concurrently. This design generally does not allow proof of causality because the causal sequence may remain unclear. However, the observed association between these *PSCA* polymorphisms and risk of DU/GU is likely relatively valid, given that genetic polymorphisms are determined by nature. Second, as the participants in this study were recruited from selected areas, they may have differed from the general population. However, the equivalence of genotype distributions between our subjects and those in another Japanese database indicates a lack of such bias. Moreover, our analysis considered study site in the models, which also reduces the likelihood of such bias. Third, we assessed *H. pylori* status for only some subjects, and the evaluation may be insufficient. However, many previous studies reported a lack of association between *PSCA* rs2294008 and *H. pylori* infection prevalence.^9^^,^^31^^–^^34^ and Hishida et al also reported a lack of association in this population.^35^ Our result is in accordance with this finding. Thus, *H. pylori* infection is less likely to bias the association between *PSCA* rs2294008 and DU/GU risk. Fourth, non-steroidal anti-inflammatory drugs and psychological stress are also known risk factors of peptic ulcer,^1^ but we did not consider these variables in our analysis due to their lack of inclusion in the questionnaire. Further analysis which considers them would be informative. Finally, as the information about past medical history was collected from the questionnaire, it might have been affected by information bias. However, participants answered the questionnaire without knowledge of their genotype, making information bias unlikely; moreover, if any misclassification were present, it would likely be nondifferential and, therefore, likely to underestimate the causal association.

In conclusion, this study confirms an association between the *PSCA* rs2294008 C-allele and risk of DU in the Japanese population. This association was independent of age, sex, study site, smoking habit, drinking habit, coffee consumption, and *H. pylori* status. Further studies examining the biological mechanism behind these associations is required.

## APPENDIX A. SUPPLEMENTARY DATA

eFigure 1. SNP selection for the *PSCA* gene. We selected *PSCA* 6 SNPs based on HapMap-JPT data using the National Institute of Health (NIH) LD TAG SNP Selection. We applied an R^2^ threshold of 0.8 for SNPs with an MAF more than 0.05.

eFigure 2. SNP selection for the *ABO* gene. We selected *ABO* 18 SNPs based on HapMap-JPT data using the National Institute of Health (NIH) LD TAG SNP Selection. We applied an R^2^ threshold of 0.8 for SNPs with an MAF more than 0.05.

eTable 1. Allele frequencies of *PSCA* and *ABO* SNPs at survey

eTable 2. Association of *PSCA* and *ABO* SNPs with risk of duodenal ulcer and gastric ulcer (analysis of data of allele dosage imputed by Minimac3)

eTable 3. Association of *PSCA* and *ABO* SNPs with risk of *H. pylori* infection
