# Supplementary material for: Impact of PSCA Polymorphisms on the Risk of Duodenal Ulcer
Source: J Epidemiol. 2021 Jan 5;31(1):12–20. doi: 10.2188/jea.JE20190184 (PMC7738644; doi:10.2188/jea.JE20190184)
Supplement: Supplementary file 1 [file je-31-012-s001.pdf]

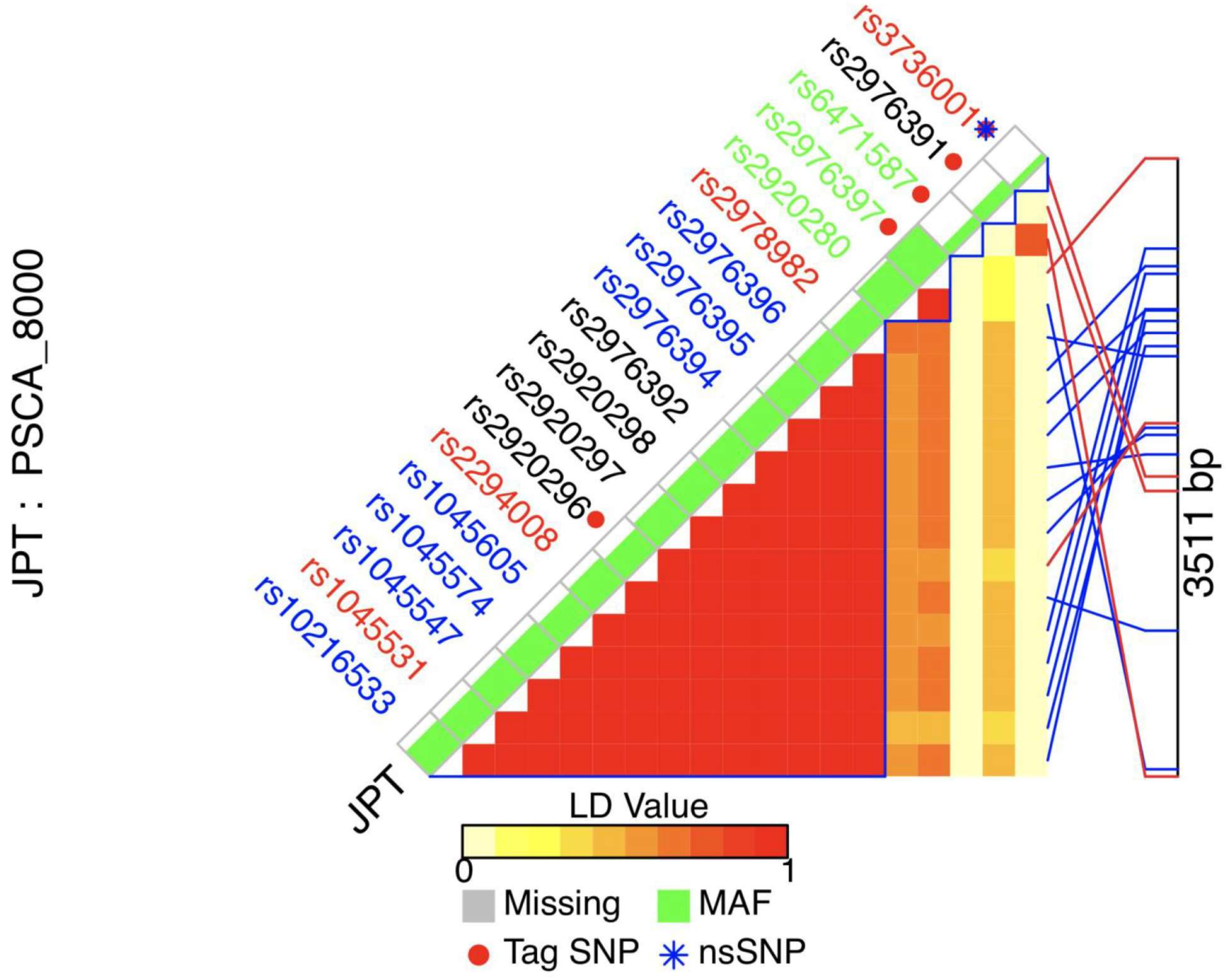

eFigure 1. SNP selection for the *PSCA* gene. We selected *PSCA* 6 SNPs based on HapMap-JPT data using the National Institute of Health (NIH) LD TAG SNP Selection. We applied an R^2 threshold of 0.8 for SNPs with an MAF more than 0.05.

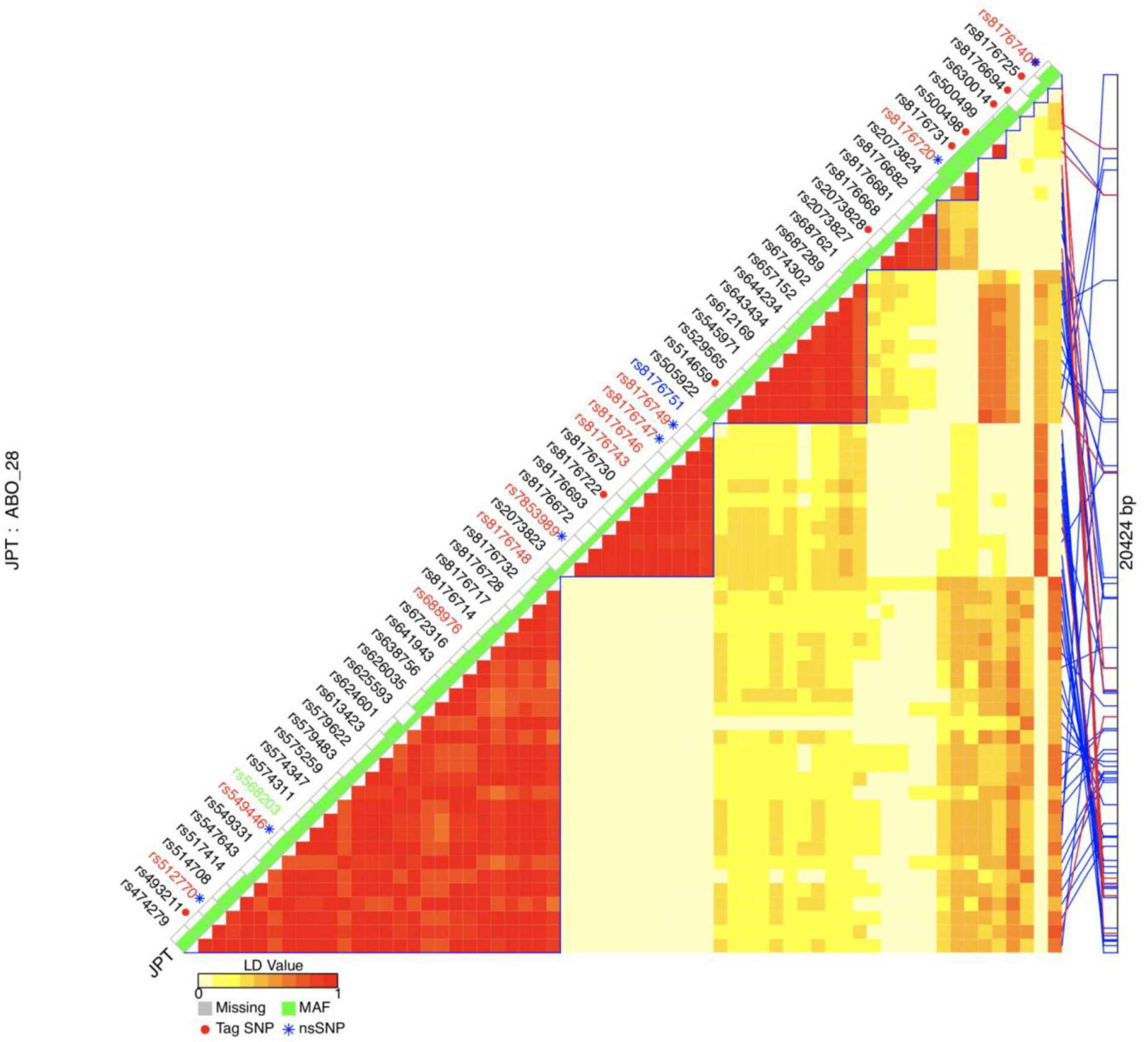

eFigure 2. SNP selection for the *ABO* gene. We selected *ABO* 18 SNPs based on HapMap-JPT data using the National Institute of Health (NIH) LD TAG SNP Selection. We applied an R^2 threshold of 0.8 for SNPs with an MAF more than 0.05.

eTable 1. Allele frequencies of *PSCA* and *ABO* SNPs at survey

					Total subjects									MAF in HapMap JPT[d]	MAF in HGVD[e]	MAF in IJGVD[f]
					n			Frequency			P value for HWE[b]	R^2 at imputation	MAF[c]			
Gene	rs number	Chr	Position	Allele A/a[a]	AA	Aa	aa	AA	Aa	aa						
PSCA	rs6471587	8q24	143761103	C/G	7127	2319	168	0.741	0.241	0.018	0.190	0.964	0.138	0.136	NA	0.141
PSCA	rs2294008	8q24	143761931	T/C	3493	4558	1563	0.363	0.474	0.163	0.240	0.854	0.400	0.378	0.379	0.357
PSCA	rs2976391	8q24	143762724	C/A	6261	2983	370	0.651	0.310	0.039	0.532	0.914	0.194	0.182	NA	0.184
PSCA	rs3736001	8q24	143762807	G/A	7713	1798	103	0.802	0.187	0.011	0.876	0.999	0.104	0.089	0.118	0.107
PSCA	rs2920296	8q24	143763109	G/A	3487	4564	1563	0.363	0.475	0.163	0.283	0.864	0.400	0.329	NA	0.358
PSCA	rs2976397	8q24	143764613	G/T	2825	4739	2050	0.294	0.493	0.213	0.450	0.877	0.460	0.500	0.476	0.493
ABO	rs8176749	9q34	136131188	C/T	6661	2690	263	0.693	0.280	0.027	0.663	0.999	0.167	0.174	0.164	0.166
ABO	rs8176747	9q34	136131315	C/G	6565	2757	292	0.683	0.287	0.030	0.902	1.000	0.174	0.163	NA	0.162
ABO	rs8176740	9q34	136131472	A/T	5199	3734	681	0.541	0.388	0.071	0.765	1.000	0.265	0.375	0.260	0.262
ABO	rs7853989	9q34	136131592	G/C	6503	2803	308	0.676	0.292	0.032	0.779	1.000	0.178	0.193	0.168	0.176
ABO	rs8176731	9q34	136132350	T/C	2958	4723	1933	0.308	0.491	0.201	0.545	0.999	0.447	0.500	NA	0.437
ABO	rs8176725	9q34	136132617	G/A	5142	3768	704	0.535	0.392	0.073	0.704	1.000	0.269	0.250	0.296	0.257
ABO	rs8176722	9q34	136132754	C/A	6449	2850	315	0.671	0.296	0.033	0.995	1.000	0.181	0.186	0.178	0.180
ABO	rs8176720	9q34	136132873	T/C	2992	4711	1911	0.311	0.490	0.199	0.467	1.000	0.444	0.506	0.428	0.444
ABO	rs512770	9q34	136133506	G/A	5238	3699	677	0.545	0.385	0.070	0.492	0.999	0.263	0.320	0.243	0.267
ABO	rs549446	9q34	136135238	C/T	5205	3717	692	0.541	0.387	0.072	0.421	1.000	0.265	0.333	0.247	0.517
ABO	rs493211	9q34	136136516	G/A	5205	3718	691	0.541	0.387	0.072	0.444	0.998	0.265	0.314	0.262	0.275
ABO	rs688976	9q34	136136770	C/A	5205	3717	692	0.541	0.387	0.072	0.421	1.000	0.265	0.326	0.247	0.275
ABO	rs2073828	9q34	136137140	G/A	5036	3789	789	0.524	0.394	0.082	0.043	1.000	0.279	0.279	0.294	NA
ABO	rs8176694	9q34	136137646	T/C	7471	1998	145	0.777	0.208	0.015	0.388	0.999	0.119	0.157	0.099	0.124
ABO	rs514659	9q34	136142203	A/C	2950	4663	2001	0.307	0.485	0.208	0.045	1.000	0.451	0.361	0.467	NA
ABO	rs500498	9q34	136148647	C/T	2957	4671	1986	0.308	0.486	0.207	0.073	0.996	0.450	0.517	NA	NA
ABO	rs505922	9q34	136149229	T/C	2884	4690	2040	0.300	0.488	0.212	0.100	1.000	0.456	0.395	0.459	NA
ABO	rs630014	9q34	136149722	G/A	3796	4492	1326	0.395	0.467	0.138	0.960	1.000	0.372	0.436	0.363	0.382

[a]Allele A, major allele; allele a, minor allele

[b]HWE: Hardy-Weinberg Equilibrium. P values <2.08E-03 (0.05/24) are highlighted in boldface.

[c]MAF: minor allele frequency.

[d]We obtained MAF in HapMap JPT from SNP annotation tool SNP nexus (http://snp-nexus.org). NA; not available.

[e]We obtained MAF in Japanese from Human Genetic Variation Database (HGVD) (https://ijgvd.megabank.tohoku.ac.jp/). NA; not available.

[f]We obtained MAF in Japanese from Integrative Japanese Genome Variation Database (IJGVD) (http://www.hgvd.genome.med.kyoto-u.ac.jp). NA; not available.

eTable 2. Association of *PSCA* and *ABO* SNPs with risk of duodenal ulcer and gastric ulcer (analysis of data of allele dosage imputed by Minimac3)

Gene	rs number	Chr	Position	Allele A/a[a]	Duodenal ulcer			Gastric ulcer		
					Per allele OR[b]	95% CI[b]	P value[b]	Per allele OR[b]	95% CI[b]	P value[b]
PSCA	rs6471587	8q24	143761103	C/G	0.96	0.80 - 1.15	6.87E-01	1.01	0.87 - 1.16	9.16E-01
PSCA	rs2294008	8q24	143761931	T/C	1.35	1.18 - 1.53	**6.08E-06**	1.08	0.97 - 1.20	1.80E-01
PSCA	rs2976391	8q24	143762724	C/A	1.24	1.07 - 1.44	4.52E-03	0.98	0.86 - 1.11	7.45E-01
PSCA	rs3736001	8q24	143762807	G/A	1.01	0.83 - 1.23	9.07E-01	0.99	0.84 - 1.16	8.69E-01
PSCA	rs2920296	8q24	143763109	G/A	1.35	1.19 - 1.53	**5.37E-06**	1.08	0.97 - 1.20	1.76E-01
PSCA	rs2976397	8q24	143764613	G/T	0.75	0.66 - 0.86	**2.10E-05**	0.93	0.83 - 1.03	1.58E-01
ABO	rs8176749	9q34	136131188	C/T	1.03	0.88 - 1.21	7.03E-01	0.89	0.78 - 1.02	1.05E-01
ABO	rs8176747	9q34	136131315	C/G	1.03	0.88 - 1.21	6.82E-01	0.88	0.77 - 1.01	6.47E-02
ABO	rs8176740	9q34	136131472	A/T	0.99	0.86 - 1.13	8.89E-01	0.95	0.85 - 1.07	4.20E-01
ABO	rs7853989	9q34	136131592	G/C	1.03	0.88 - 1.20	7.31E-01	0.89	0.78 - 1.01	7.45E-02
ABO	rs8176731	9q34	136132350	T/C	1.01	0.90 - 1.14	8.21E-01	0.90	0.82 - 0.99	3.85E-02
ABO	rs8176725	9q34	136132617	G/A	1.00	0.88 - 1.15	9.70E-01	0.93	0.83 - 1.04	1.79E-01
ABO	rs8176722	9q34	136132754	C/A	1.04	0.89 - 1.21	6.24E-01	0.89	0.78 - 1.01	7.25E-02
ABO	rs8176720	9q34	136132873	T/C	1.01	0.90 - 1.14	8.60E-01	0.89	0.81 - 0.98	2.05E-02
ABO	rs512770	9q34	136133506	G/A	0.98	0.86 - 1.13	8.25E-01	0.94	0.84 - 1.05	2.91E-01
ABO	rs549446	9q34	136135238	C/T	0.99	0.86 - 1.13	8.28E-01	0.93	0.84 - 1.05	2.40E-01
ABO	rs493211	9q34	136136516	G/A	0.99	0.86 - 1.13	8.33E-01	0.94	0.84 - 1.05	2.43E-01
ABO	rs688976	9q34	136136770	C/A	0.98	0.86 - 1.13	8.07E-01	0.94	0.84 - 1.05	2.41E-01
ABO	rs2073828	9q34	136137140	G/A	1.01	0.89 - 1.16	8.27E-01	1.07	0.96 - 1.19	2.40E-01
ABO	rs8176694	9q34	136137646	T/C	0.98	0.82 - 1.18	8.65E-01	0.88	0.75 - 1.03	1.18E-01
ABO	rs514659	9q34	136142203	A/C	1.01	0.89 - 1.14	8.96E-01	0.99	0.90 - 1.10	8.97E-01
ABO	rs500498	9q34	136148647	C/T	0.94	0.84 - 1.06	3.45E-01	0.97	0.87 - 1.06	4.79E-01
ABO	rs505922	9q34	136149229	T/C	1.02	0.90 - 1.15	7.94E-01	0.99	0.89 - 1.09	7.93E-01
ABO	rs630014	9q34	136149722	G/A	1.00	0.88 - 1.13	9.90E-01	1.01	0.91 - 1.12	8.33E-01

[a]Allele A, major allele; allele a, minor allele

[b]For additive models, gender-, age- and site-adjusted per allele OR; 95% CI and P values calculated by logistic regression are shown. Major alleles were considered as references. P values <2.08E-03 (0.05/24) are highlighted in boldface. Threshold was Bonferroni significance.

eTable 3. Association of *PSCA* and *ABO* SNPs with risk of *H. pylori* infection

					H. pylori status								
					Negative (n=1,787)			Positive (n=973)					
					Genotype prevalence			Genotype prevalence					
Gene	rs number	Chr	Position	Allele A/a[a]	AA	Aa	aa	AA	Aa	aa	Per allele OR[b]	95% CI[b]	P value[b]
PSCA	rs6471587	8q24	143761103	C/G	0.722	0.259	0.020	0.726	0.259	0.015	0.95	0.80 - 1.12	5.16E-01
PSCA	rs2294008	8q24	143761931	T/C	0.363	0.467	0.171	0.356	0.456	0.188	1.03	0.92 - 1.15	6.12E-01
PSCA	rs2976391	8q24	143762724	C/A	0.634	0.322	0.045	0.655	0.306	0.039	0.90	0.78 - 1.04	1.66E-01
PSCA	rs3736001	8q24	143762807	G/A	0.781	0.206	0.013	0.783	0.205	0.012	0.97	0.81 - 1.16	7.59E-01
PSCA	rs2920296	8q24	143763109	G/A	0.361	0.468	0.171	0.355	0.459	0.186	1.02	0.91 - 1.15	6.88E-01
PSCA	rs2976397	8q24	143764613	G/T	0.313	0.494	0.194	0.323	0.481	0.196	1.02	0.91 - 1.14	7.42E-01
ABO	rs8176749	9q34	136131188	C/T	0.705	0.267	0.028	0.676	0.288	0.036	1.18	1.02 - 1.37	2.97E-02
ABO	rs8176747	9q34	136131315	C/G	0.697	0.271	0.031	0.669	0.293	0.038	1.16	1.00 - 1.35	4.87E-02
ABO	rs8176740	9q34	136131472	A/T	0.562	0.373	0.066	0.577	0.366	0.058	0.94	0.82 - 1.07	3.31E-01
ABO	rs7853989	9q34	136131592	G/C	0.695	0.273	0.319	0.667	0.293	0.040	1.16	1.00 - 1.35	4.28E-02
ABO	rs8176731	9q34	136132350	T/C	0.333	0.487	0.180	0.328	0.475	0.197	1.06	0.94 - 1.19	3.38E-01
ABO	rs8176725	9q34	136132617	G/A	0.552	0.378	0.070	0.503	0.411	0.086	1.21	1.06 - 1.37	3.83E-03
ABO	rs8176722	9q34	136132754	C/A	0.691	0.276	0.033	0.654	0.306	0.040	1.19	1.03 - 1.38	2.09E-02
ABO	rs8176720	9q34	136132873	T/C	0.337	0.486	0.177	0.332	0.474	0.194	1.06	0.94 - 1.19	3.48E-01
ABO	rs512770	9q34	136133506	G/A	0.566	0.371	0.064	0.581	0.363	0.057	0.93	0.82 - 1.07	3.15E-01
ABO	rs549446	9q34	136135238	C/T	0.562	0.373	0.064	0.579	0.364	0.058	0.93	0.82 - 1.06	2.99E-01
ABO	rs493211	9q34	136136516	G/A	0.562	0.373	0.064	0.579	0.364	0.058	0.93	0.82 - 1.06	2.99E-01
ABO	rs688976	9q34	136136770	C/A	0.562	0.373	0.064	0.579	0.364	0.058	0.93	0.82 - 1.06	2.99E-01
ABO	rs2073828	9q34	136137140	G/A	0.501	0.412	0.087	0.511	0.391	0.099	0.98	0.86 - 1.11	7.02E-01
ABO	rs8176694	9q34	136137646	T/C	0.800	0.184	0.016	0.797	0.195	0.008	0.99	0.83 - 1.19	9.43E-01
ABO	rs514659	9q34	136142203	A/C	0.312	0.473	0.215	0.304	0.475	0.221	1.06	0.94 - 1.18	3.44E-01
ABO	rs500498	9q34	136148647	C/T	0.314	0.483	0.203	0.309	0.496	0.194	0.97	0.86 - 1.09	5.91E-01
ABO	rs505922	9q34	136149229	T/C	0.310	0.471	0.219	0.301	0.478	0.221	1.05	0.94 - 1.18	3.74E-01
ABO	rs630014	9q34	136149722	G/A	0.413	0.449	0.138	0.421	0.446	0.133	0.96	0.85 - 1.08	4.53E-01

[a]Allele A, major allele; allele a, minor allele

[b]For additive models, gender-, age- and site-adjusted per allele OR; 95% CI and P values calculated by logistic regression are shown.
Major alleles were considered as references. P values <2.08E-03 (0.05/24) are highlighted in boldface. Threshold was Bonferroni significance.
